# Compartmentalization and Transport in Synthetic Vesicles

**DOI:** 10.3389/fbioe.2016.00019

**Published:** 2016-02-29

**Authors:** Christine Schmitt, Anna H. Lippert, Navid Bonakdar, Vahid Sandoghdar, Lars M. Voll

**Affiliations:** ^1^Division of Biochemistry, Friedrich-Alexander-Universität Erlangen-Nürnberg, Erlangen, Germany; ^2^Max-Planck-Institute for the Science of Light, Erlangen, Germany

**Keywords:** liposomes, vesosomes, block copolymers, reconstitution techniques, porins, metabolite transporters, membrane transport, compartmentalized bioreactors

## Abstract

Nanoscale vesicles have become a popular tool in life sciences. Besides liposomes that are generated from phospholipids of natural origin, polymersomes fabricated of synthetic block copolymers enjoy increasing popularity, as they represent more versatile membrane building blocks that can be selected based on their specific physicochemical properties, such as permeability, stability, or chemical reactivity. In this review, we focus on the application of simple and nested artificial vesicles in synthetic biology. First, we provide an introduction into the utilization of multicompartmented vesosomes as compartmentalized nanoscale bioreactors. In the bottom-up development of protocells from vesicular nanoreactors, the specific exchange of pathway intermediates across compartment boundaries represents a bottleneck for future studies. To date, most compartmented bioreactors rely on unspecific exchange of substrates and products. This is either based on changes in permeability of the coblock polymer shell by physicochemical triggers or by the incorporation of unspecific porin proteins into the vesicle membrane. Since the incorporation of membrane transport proteins into simple and nested artificial vesicles offers the potential for specific exchange of substances between subcompartments, it opens new vistas in the design of protocells. Therefore, we devote the main part of the review to summarize the technical advances in the use of phospholipids and block copolymers for the reconstitution of membrane proteins.

## Introduction

Compartmentalization is a key feature of eukaryotic cells to spatially separate distinct biochemical processes from each other. Lipid bilayer membranes serve as impermeable barriers that effectively separate subcellular compartments. This (i) enables the simultaneous operation of metabolic pathways that utilize the same intermediates and (ii) allows for the adjustment of specific reaction conditions inside individual organelles. In order to translate this natural principle of biological organization, the use of membranes to encapsulate chemical reactions has attracted interest in the design of synthetic systems. Lipids or block copolymers are commonly used to build membranes in synthetic systems, in so-called liposomes or polymersomes, respectively.

**Figure 1:**
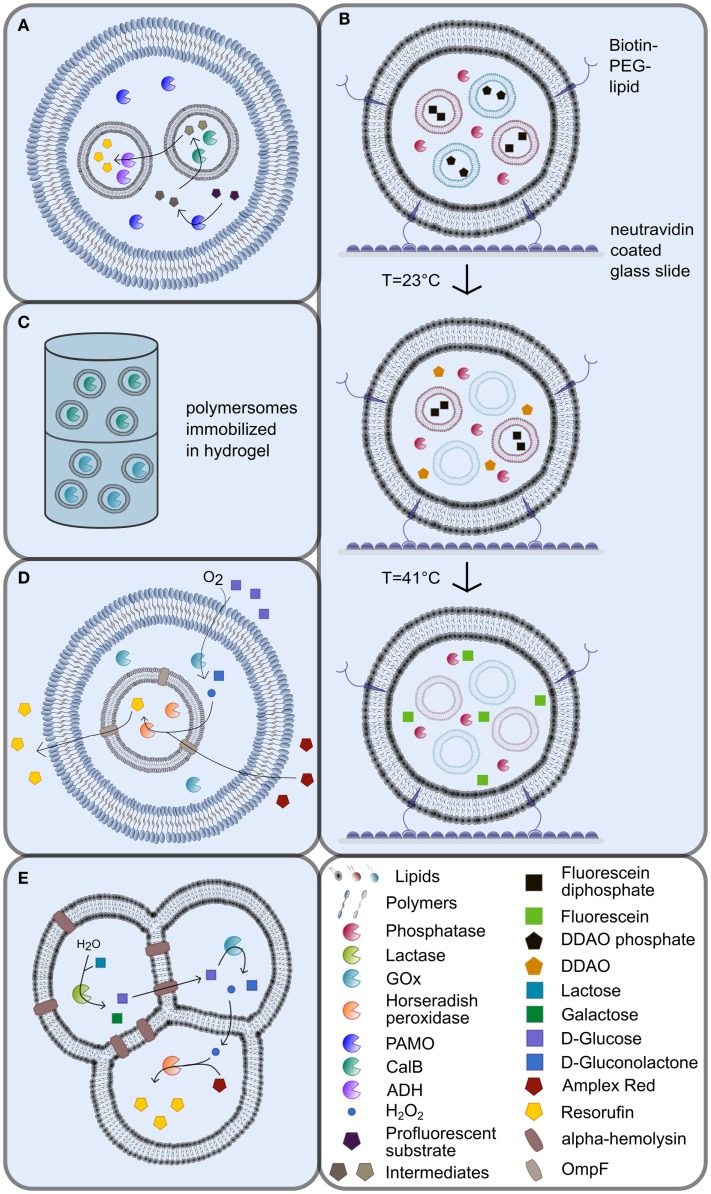
**Multicompartmented artificial vesicles as bioreactors**. **(A)** Vesosomes consisting of inner and outer coblock polymersomes with different physicochemical properties [adapted from (Peters et al., 2014)]. **(B)** Immobilization of biotinylated lipid vesosomes *via* the interaction with neutravidin in nanofluidic reactors [adapted from (Bolinger et al., 2008)]. The release of liposome contents is triggered by specific, consecutive temperature shifts. **(C)** Continuous-flow polymersome reactor with immobilized polymersomes in hydrogel (De Hoog et al., 2010). **(D)** Vesosomes using the porin OmpF as shuttle system [adapted from (Siti et al., 2014)]. **(E)** Multicompartment liposomes generated by the phase transfer technique [adapted from (Elani et al., 2014)]. For explanations, please refer to the manuscript text.

Liposomes consist of a shell of amphiphilic lipid species, such as phospholipids, that encapsulate an aqueous solution. The lipids are arranged in a bilayer with the polar head groups of the two leaflets facing toward the inside and the outside aqueous phase and the hydrophobic tails of the phospholipids facing toward each other (Figure 1B). Based on the number of membrane layers, vesicles are called unilamellar or multilamellar. For biotechnological applications, the use of unilamellar vesicles is desirable, and these vesicle species can be defined according to their size, ranging from small unilamellar vesicles (SUVs having a diameter between 25 and 100 nm) to large unilamellar vesicles (LUVs with a diameter between 100 nm and 1 μm) and giant unilamellar vesicles (GUVs being larger than 1 μm up to 100 μm in diameter). Polymersomes consisting of amphiphilic block copolymers are of rising interest for compartmentalization in synthetic systems due to their increased mechanical stability and low membrane permeability compared to liposomes. The ability to (i) encapsulate specific cargo, (ii) trigger cargo release by external stimuli, and (iii) the possibility to incorporate particular membrane transport proteins are distinct features that depend on liposome and polymersome composition of artificial vesicles.

## The Potential of Liposomes, Polymersomes, and Vesosomes – An Overview

Both liposomes and polymersomes have become popular as vectors for targeted and tailored drug delivery and for the application in biochemical microreactors. Due to the amphiphilic nature of the lipid or polymer building blocks, a spontaneous assembly into vesicles occurs in aqueous environments (Discher and Eisenberg, 2002). The phase transition temperature represents an important parameter for the choice of specific lipid or polymer building blocks for drug delivery purposes. At the phase transition temperature, lipids and polymers are transformed from a liquid crystalline phase to a gel phase, which leads to maximal bilayer permeability (Van Hoogevest et al., 1984), and hence, to the release of cargo from the lumen of the vesicles. Based on the choice of lipid or block copolymer, the discharge of cargo from artificial vesicles can also be achieved by pH shifts, or redox potential, which we will discuss later in this article. However, the use of artificial vesicles for drug delivery is out of focus of this review, and we would like to refer the interested reader to recent reviews focusing on this topic (Ohya et al., 2011; Lee and Feijen, 2012; Khan et al., 2015; Thambi et al., 2016).

For vesicle formation, a variety of lipids with different properties is available (Marsh, 2012), which can either be used separately or as mixtures. Polymersomes made of amphiphilic block copolymers came into focus because of their potential for functionalization and increased mechanical stability compared to liposomes (Bermudez et al., 2002). Polyethylene glycol (PEG) and polyesytrene (PS)-based block copolymers are widely used to produce polymersomes for all kinds of applications. In addition, polypeptide-based polymersomes have become increasingly popular for biomedical applications, which are not only due to their biodegradability and high tissue compatibility but also based on their ability to change aggregation state and permeability in response to environmental stimuli [as recently reviewed by Zhao et al. (2014)]. Membrane thickness of copolymer-derived polymersomes predominantly depends on the length of the hydrophobic block (Smart et al., 2008). However, not only the chain length of the individual hydrophilic and hydrophobic blocks in diblock and triblock copolymers but also the length ratio of the hydrophilic and hydrophobic segments were found to represent an important parameter for membrane permeability and stiffness (Rodríguez-García et al., 2011). Copolymers that combine a low molecular weight with high hydrophobicity were found to preferably arrange into GUVs (Rodríguez-García et al., 2011). For a more in depth view on the use of polymersomes as vesicle scaffolds in biotechnology, please see recent reviews on the topic (e.g., Lee and Feijen, 2012; Zhao et al., 2014).

Besides simple, single-compartment vesicles, the formation of multicompartmentalized vesicular systems was engineered in the last years to allow the encapsulation of distinct cargos in different vesicular compartments. Various approaches were investigated for this purpose, such as the encapsulation of smaller vesicles into larger vesicles, so-called vesosomes (Walker et al., 1997; Bolinger et al., 2008; Marguet et al., 2012; Paleos et al., 2012). Vesosomes represent nested vesicles that harbor multiple compartments of different sizes encapsulated in each other without a direct connection between the individual compartment boundaries (Figures 1A,B,D).

## Vesicles and Vesosomes as Compartmentalized Nanoreactors

Compartmentation of enzymatic reactions represents the basic biochemical principle of all living systems. The compartmentation of cellular metabolism has many advantages: the cell can be protected against toxic intermediates formed in one compartment, cellular subcompartments offer optimal reaction conditions for subsets of enzymes, compartmentation can avoid competition for substrates by different metabolic pathways, and it permits the differential regulation of isoenzymes within distinct compartments. Therefore, it seems reasonable to attempt to design multicompartmentalized synthetic systems that are confined by lipid or polymer bilayers, which provide optimal reaction conditions for different enzyme species. Pioneering work in the field was obtained when Meier and coworkers reported an enzymatic reaction inside a PMOXA–PDMS–PMOXA triblock copolymer vesicle in 2000. Passive diffusion of the substrate ampicillin into the polymersome was mediated by the reconstituted bacterial porin OmpF, the encapsulated enzyme β-lactamase subsequently hydrolyzed the substrate before the product ampicillinoic acid diffused out again through OmpF (Nardin et al., 2000, 2001).

To date, vesosomes have successfully been applied to generate compartmentalized biochemical systems, e.g., in the co-factor-dependent enzymatic formation of the fluorescent dye resorufin from a profluorescent substrate (Peters et al., 2014). To this end, the two different enzyme species *Candida antarctica* lipase B (CalB) and alcohol dehydrogenase (ADH) were encapsulated separately into intrinsically porous sub-micrometer-sized PS-*b*–PIAT polymersome subcompartments, which, in turn, were engulfed by PB-*b*-PEO GUVs (Figure 1A). In such a vesosome assembly, the lumen of the PB-*b*-PEO vesicle resembles an artificial cytosol, while the PS-*b*–PIAT vesicle lumen corresponds to artificial organelles (Figure 1A). In the initial step, the substrate was converted by NADPH-dependent phenylacetone monooxygenase (PAMO) into an ester in the artificial cytosol of the vesosomes, before the ester intermediate diffused into the CalB containing subcompartments, where it was subsequently hydrolyzed to a primary alcohol. After diffusion of the alcohol product out of the first subcompartment into the ADH containing subcompartments, the alcohol was oxidized in a NAD^+^-dependent reaction into an aldehyde, which then produced the fluorescent dye resorufin by spontaneous beta-elimination (Figure 1A). Likewise, other cascade reactions have also been established in polymersomes using glucose oxidase (GOx), horse radish peroxidase (HRP), and CalB (Vriezema et al., 2007; Kuiper et al., 2008).

Even complex cellular processes, such as the synthesis of ATP, could be achieved by the coupled activity of bacteriorhodopsin and F_0_F_1_-ATP synthase in synthetic vesicles. An H^+^ gradient was built up by bacteriorhodopsin in a light-dependent manner and this H^+^ gradient was subsequently utilized by ATP synthase to convert ADP and P_i_ to ATP. These two membrane-associated proteins have successfully been reconstituted into amphiphilic triblock copolymer PEtOz-PDMS-PEtOz polymersomes leading to ATP synthesis (Choi and Montemagno, 2005). This example nicely demonstrates the potential of synthetic systems to mimic complex cellular functions.

The potential to immobilize vesicular systems can also be exploited for the application as nanoreactors in nanofluidic devices, since the provision of substrate to and the harvest of product from immobilized vesicular compartments is much easier compared to open reaction systems. To this end, vesosomes have been immobilized on neutravidin-coated glass surfaces in nanoreactor systems through the integration of biotin–PEG–lipids into the outer vesosome bilayer [Figure 1B; Bolinger et al. (2008)]. In these systems, the inner SUVs consisted of lipids with different phase transition temperatures compared to the outer SUVs. The inner SUVs were loaded with the profluorescent dyes dichlorodimethylacridinone phosphate or fluorescein diphosphate, while the outer compartment was loaded with alkaline phosphatase (AP). The sequential, temperature-triggered release of the substrates from the encapsulated SUVs drove the conversion of the substrates by AP in the outer compartment in two distinct, consecutive steps (Figure 1B). The produced fluorescent products dichlorodimethylacridinone and fluorescein, respectively, were still trapped inside the outer lipid bilayer.

Alternatively, artificial vesicles have been immobilized in alginate capsules or hydrogels (De Hoog et al., 2010; Ullrich et al., 2015). A “continuous-flow polymersome reactor” was constructed by the immobilization of CalB and GOx loaded polymersomes in a hydrogel (Figure 1C) (De Hoog et al., 2010). The substrate was added on top of the reactor in this setup, while the product was collected at the bottom (Figure 1C). Since enzyme leakage from immobilized polymersomes was more than four times lower compared to free enzyme, the total enzyme activity required for nanoreactors can be decreased, once the proteins are encapsulated into polymersomes (De Hoog et al., 2010). These two examples illustrate the advantages of vesicular functional units in nanoreactor assemblies.

## Controlled Release of Cargo from Liposomes, Polymersomes, and Vesosomes by Physicochemical Triggers

An important feature in the construction of vesicle-based nanoreactors is the design of the vesicle shell by the choice of lipids, polymers, or a mixture of both. Based on the chosen lipid or block copolymer, a specific release of cargo by external stimuli can be achieved following a physical or chemical trigger that alters membrane permeability. Before we focus on membrane transport proteins for the specific exchange of solutes between vesicle compartments, we would like to briefly summarize the advances in the use of block copolymers that permit a triggered release of solutes.

The release of ABTS^2−^ by repeated thermal stimuli and the subsequent conversion to ABTS^1−^ by laccase inside the alginate capsules was observed by Ullrich et al. (2015). The heat stimulus was applied either by heating above the phase transition temperature of DPPC in a water bath or by subjecting encapsulated superparamagnetic iron oxide nanoparticles to radiofrequency to cause heat emission.

In addition, stimuli- and cargo-selective content release was achieved in dual stimuli-responsive polymersomes with two kinds of cargo (Staff et al., 2014). On the one hand, the polymer vesicles consisted of the redox- and pH-responsive polymer polyvinylferrocene-*b*-poly(2-vinylpyridine) (PVFc-*b*-P2VP) or the pH- and temperature-responsive polymer polystyrene-*b*-poly(*N*,*N*-dimethylaminoethyl methacrylate) (PS-*b*-PDMAEMA). On the other hand, the cargos dimethyldodecylamine (DDA) and diphenyl disulfide (DPDS) were selectively switchable from the water-insoluble to the soluble form by pH change or H_2_O_2_ redox trigger, respectively. The substances were stored in the water-insoluble form inside the polymersomes and were then specifically released by an external stimulus that allowed the passage of DPDS (by oxidation) or DDA (by pH change) across the membrane.

Finally, vesosomes were composed of lipids with different phase transition temperatures were used to trigger the successive mixing of the contents inside the vesosome by a suite of specific temperature changes (Bolinger et al., 2004, 2008). Likewise, the incorporation of stimuli-responsive polymers into polymersomes, such as the sugar and pH-responsive PEG-*b*-PSBA block copolymer or pH-responsive non-ionic amphiphilic triblock copolymers such as PEO-PPO-PEO, can lead to partial permeabilization of these vesicles. Pore-like structures are formed upon applying an appropriate external stimulus, but the vesicles are not disrupted (Binder, 2008; Kim et al., 2009).

## The Use of Transport Proteins for Exchange of Substrate between Vesicular Subcompartments

While the release of cargo by triggered permeabilization of the bilayer is accompanied by at least partial or temporal loss of compartmentalization, a more controlled discharge of cargo from vesicles can be achieved by the integration of membrane proteins into the vesicle membrane. This can either be achieved by the reconstitution of unspecific diffusion pores, such as porins, or by the integration of substrate-specific transporters. This strategy avoids the increase in bilayer permeability by external stimuli but instead enables specific substrate flow across compartment boundaries. A frequently used protein for this purpose is the *Escherichia coli* porin OmpF (outer membrane protein F), which is a trimeric integral membrane protein that enhances the passive diffusion of small hydrophilic molecules (Cowan et al., 1995).

The integration of OmpF in a lipid bilayer encapsulating β-lactamase inside the vesicle lead to hydrolysis of the externally added substrate ampicillin and yielded the product ampicillinoic acid (Graff et al., 2001). The product was first detected inside the vesicle before its accumulation in the medium occurred, which was also facilitated by OmpF. Since the diffusion across the membrane bilayer represented the bottleneck of the reaction, a higher substrate concentration was necessary to achieve comparable activity of encapsulated enzymes compared to free enzymes. Recently, OmpF was also reconstituted into an ABA triblock copolymer bilayer that served as the inner compartment of vesosomes that encapsulated horseradish peroxidase (Figure 1D; Siti et al., 2014). The semi-permeable outer compartment was built of PS-PIAT diblock copolymers and contained GOx as well as the inner ABA polymersomes. After glucose and Amplex Red were added to the outside solution, they diffused into the outer compartment, where glucose was oxidized by GOx to produce H_2_O_2_ (Figure 1D). Hydrogen peroxide then diffused into the inner compartment, where it subsequently oxidized Amplex Red in the presence of horseradish peroxidase to yield the fluorescent end-product Resorufin (Figure 1D). Most importantly, this study by Siti et al. (2014) showed an increased reaction rate in the presence of OmpF, which enhanced diffusion into the inner reaction compartment.

**Figure 2:**
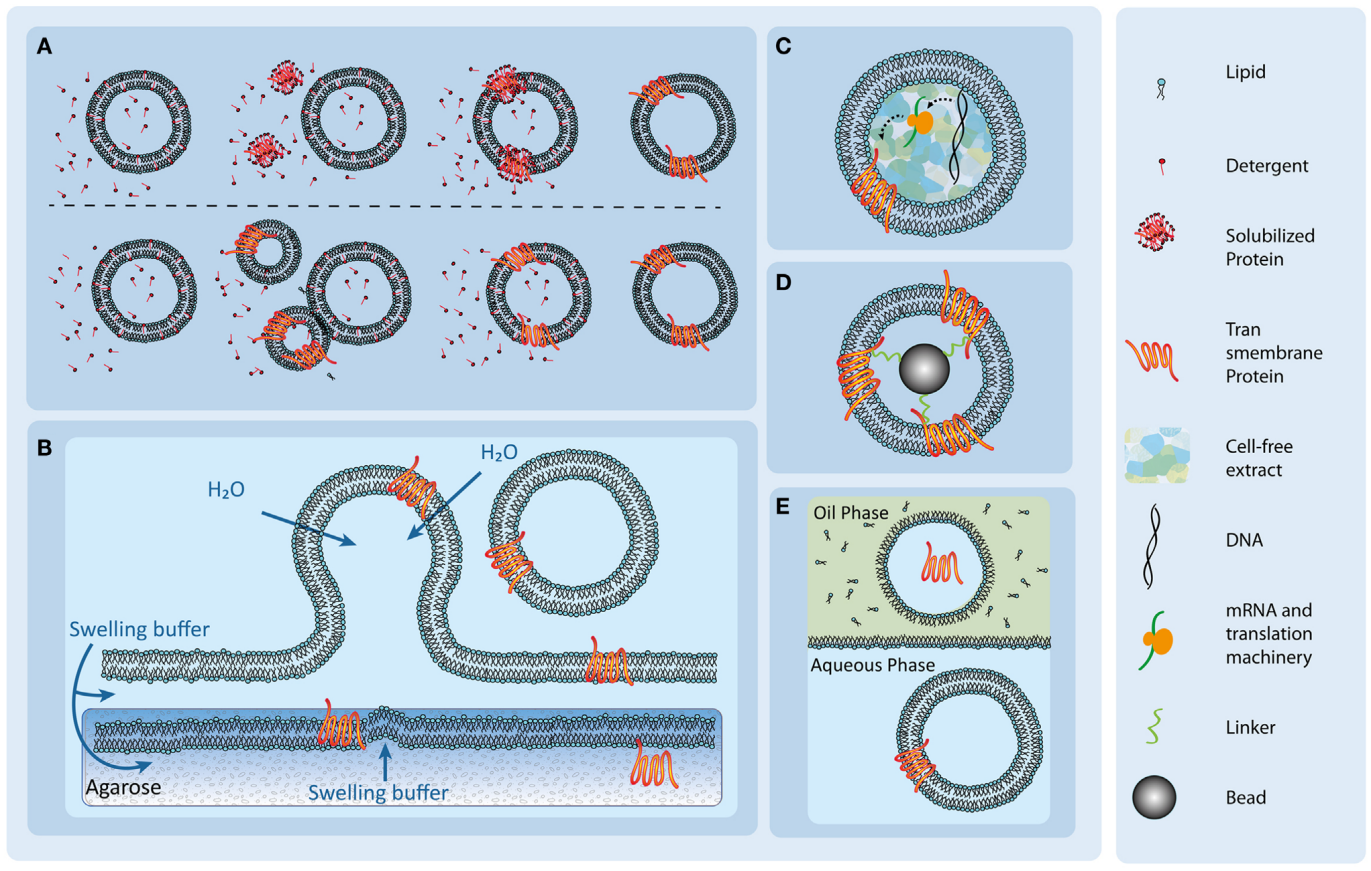
**Overview of different transmembrane protein reconstitution methods**. **(A)** Detergent-mediated protein reconstitution uses detergent molecules to trigger reconstitution (upper row) and fusion events (lower row). After successful reconstitution, the detergent molecules are removed to form stable protein-containing vesicles. Illustration adapted from Dezi et al. (2013). **(B)** Protein reconstitution *via* spontaneous swelling of vesicles from a protein-containing agarose film. The solubilized proteins are added to the agarose gels and incorporate spontaneously upon lipid addition and swelling. **(C)** An internalized cell-free extract supplied with specific DNA of the transmembrane protein leads to reconstitution of the protein in the vesicle membrane. Illustration adapted from Kaneda et al. (2009). **(D)** A proteoliposome is formed with the use of antibody or protein ligand-coated beads and the subsequent addition of lipids. Illustration adapted from Frank et al. (2015). **(E)** Spontaneous protein insertion in a double-emulsion setup. A lipid monolayer forms on the border between a lipid containing oil phase and an aqueous phase. Upon addition of an aqueous droplet, containing the solubilized protein, a micelle forms around the droplet, enclosing the solution. When the micelle passes through the lipid monolayer into the aqueous phase, a vesicle forms. The solubilized protein spontaneously inserts. Illustration adapted from Yanagisawa et al. (2011).

The constituents of the heptameric protein α-hemolysin can self-assemble in membrane bilayers to form pores, which makes it an attractive target for the use in artificial membranes. Elani et al. (2013, 2014) incorporated α-hemolysin into DOPC bilayers of multicompartment vesicle networks by phase transfer of water-in-oil droplets (Figures 1E and 2E). To proof functionality of the pore protein in a two-compartment system, the Ca^2+^-sensitive dye Fluo-4 was encapsulated in one compartment and Ca^2+^ in the other compartment. Only those vesicular systems with α-hemolysin in the internal bilayer showed an increase in fluorescence, while no fluorescence was detectable in vesicle systems that did not contain α-hemolysin (Elani et al., 2013). A spatially segregated reaction setup was established in a three-compartment system with α-hemolysin pores connecting the first compartment with the second and with the third compartment as well as with the surrounding (Figure 1E; Elani et al., 2014). Each reaction step was performed in a single compartment: in the first compartment, lactose was hydrolyzed to glucose and galactose by lactase, and glucose was then oxidized to gluconolactone in the second compartment *via* GOx, thereby producing hydrogen peroxide (Figure 1E). The diffusion of glucose from the first into the second compartment was conferred by α-hemolysin, while lipid bilayers are permeable to hydrogen peroxide to allow for the diffusion of hydrogen peroxide from the second into the third compartment (Figure 1E). Finally, hydrogen peroxide initiated the oxidation of Amplex Red by horseradish peroxidase in the third compartment to yield the fluorescent product resorufin. No increase in fluorescence was detectable in vesicular systems without α-hemolysin (Elani et al., 2014).

## Specific Transport Proteins as Tools in Artificial Vesicle Systems

The chapters above deal with the unspecific release of cargo and substrates from vesicles *via* physicochemical triggers or by unspecific porins such as OmpF or α-hemolysin. To enable specific transport of cargo and substrates, appropriate transport proteins can be reconstituted into liposome or polymersome membranes. The incorporation of specific transport proteins into artificial membranes of liposomes, polymersomes, or vesosomes allows to conceptualize much more tightly controlled vesicle-based bioreactors. Therefore, we devote the rest of this review on recent advances in the reconstitution of membrane proteins.

## Reconstitution of Transmembrane Proteins in Lipid Systems

The studies on reconstitution of transmembrane proteins into a membrane environment mainly focus on the work with liposomes (Kahya et al., 2001; Montes et al., 2007; Kaneda et al., 2009; Aimon et al., 2011; Dezi et al., 2013; Hansen et al., 2013; Liu et al., 2013) but have also been successfully applied to polymersomes (Meier et al., 2000; Choi and Montemagno, 2005; Nallani et al., 2011; Martino et al., 2012).

There are multiple ways to reconstitute transmembrane proteins in artificial membrane systems. The basic problem behind transmembrane protein reconstitution is the nature of these proteins. Since transport proteins are anchored in the hydrophobic core of the cell membrane, they have a hydrophobic nature. This hydrophobicity aggravates their extraction as well their insertion from and into membrane systems. The cell itself circumvents this problem employing several strategies (Wickner and Lodish, 1985; Gutensohn et al., 2006). One of these is the so-called Sec pathway found in bacteria and eukaryotes (Economou, 1999; Rapoport et al., 2004). Hydrophobic protein parts are co-translationally recognized by a signal recognition particle, which leads to a translocation of the protein translation machinery to the endoplasmic reticulum (ER) and the co-translational insertion of the protein into the cell membrane *via* the Sec apparatus. Another strategy involves the post-translational insertion of membrane proteins. After protein translation in the cytoplasm of eukaryotic cells, the protein is delivered, unfolded, inserted, and refolded in the target membrane, i.e., the TIC TOC (Gutensohn et al., 2006; Andrès et al., 2010) complex in the chloroplast envelope or the TOM TIM (Bauer et al., 2000) complex in mitochondria. In addition, certain transmembrane proteins exhibit spontaneous insertion into the membrane, such as predominantly cytochromes (Wickner and Lodish, 1985). It is debated whether helical hairpin motifs can mediate this spontaneous insertion of proteins into membranes (Engelman and Steitz, 1981). All in all there is a huge variety and complexity of mechanisms the cell uses to insert membrane proteins into the target membrane as well as the heterogeneity of membrane proteins involved. Similarly, there are a couple of methods available for the functional reconstitution of membrane proteins, which need to be tested for individual proteins of interest.

## Detergent-Mediated Reconstitution

Since the solubilization process of membrane proteins, the removal from their natural environment, is usually performed in the presence of detergent, the detergent-mediated reconstitution is one of the most common strategies for protein reconstitution (Figure 2A). Here, liposomes are formed *via* extrusion (Torchilin and Weissig, 2003) or sonification (Torchilin and Weissig, 2003), and after a presolubilization step, the solubilized proteins are subsequently added to the liposome preparation, which eventually leads to the incorporation of the proteins into the membranes.

After the pioneering work of Kagawa and Racker (1971), the detergent-mediated reconstitution has been successfully reported on the reconstitution of various transmembrane proteins in vesicles up to sizes larger than 1 μm (Rigaud et al., 1988; Steinberg-Yfrach et al., 1998; Seddon et al., 2004; Dezi et al., 2013). The successfully reconstituted proteins includes porins, such as FhuA (Dezi et al., 2013), transporters such as bacteriorhodopsin (Steinberg-Yfrach et al., 1998), the Glucose-6-P/P antiporter (Kammerer et al., 1998), H^+^-ATPase (Steinberg-Yfrach et al., 1998), Ca^2+^-ATPase (Steinberg-Yfrach et al., 1998), CFOF1-ATPase (Steinberg-Yfrach et al., 1998), and channels, such as the voltage-gated potassium channel (Ruta et al., 2003), BmrC/BmrD, a bacterial heterodimeric ATP-binding cassette efflux transporter (Dezi et al., 2013), as well as receptor proteins such as GPCRs (Ishihara et al., 2005).

With the advantage of very low external influences on the membrane protein, the detergent-mediated reconstitution is a suitable method for many proteins. The reconstitution of the membrane proteins is either achieved through direct incorporation of solubilized membrane proteins or, in order to form larger proteoliposomes, through detergent-mediated fusion of vesicles. In both cases, the vesicles are often pre-solubilized by detergent prior to the addition of proteins. The detergent-to-lipid ratio needs to be meticulously adjusted to achieve a fusogenic liposome state that ranges between detergent saturation and solubilization of the vesicle. For detergents, the relationship between critical micelle concentration and lipid-to-detergent ratio is given by (Ollivon et al., 2000; Rigaud and Lévy, 2003).

$D_{\text{total}}=D_{\text{water}}+R_{\text{eff}}\cdot L$, where *D*_total_ represents the total detergent concentration, *D*_water_ provides the monomeric detergent concentration in water, i.e., the cmc determined in the presence of lipids, while *L* represents the lipid concentration and *R*_eff_ the lipid-to-detergent ratio specific for the solubilization state. *D*_water_ and *R*_eff_ are constants that need to be determined for the detergent of choice (Ollivon et al., 2000; Rigaud and Lévy, 2003).

With increasing detergent concentration, the amount of incorporated detergent molecules in the vesicles increases, leading to the fragile state of saturation (Ollivon et al., 2000; Rigaud and Lévy, 2003). At this stage, further addition of detergent leads to vesicle shrinkage until full solubilization occurs (Ollivon et al., 2000; Rigaud and Lévy, 2003). The incorporation rates are dependent on the protein and detergent used (Rigaud and Lévy, 2003), and the state of solubilization also represents an important factor for the reconstitution rate and the orientation of the reconstituted protein (Rigaud et al., 1995; Rigaud and Lévy, 2003). It was shown that some proteins incorporate better in a state of low detergent incorporation, while other need complete saturation for an efficient reconstitution (Rigaud et al., 1995). The reconstitution efficiency thereby depends also on the type of detergent and protein used (Rigaud et al., 1995; Dezi et al., 2013). The method has to be tuned for the protein of interest to achieve the best fusion rate; therefore, there are various protocols available to achieve reconstitution but no specific protocol is applicable to all membrane proteins (Ollivon et al., 2000; Rigaud and Lévy, 2003).

After protein reconstitution, the detergent molecules need to be removed for stable vesicles to form (Ollivon et al., 2000; Rigaud and Lévy, 2003). The removal method and its efficiency are thereby dependent on the type of detergent (Rigaud et al., 1995; Rigaud and Lévy, 2003). Detergents with a high cmc, such as CHAPS, chapso, cholate, and octyl glucoside, generally form small micelles, which makes them easy to remove *via* dialysis or gel filtration (Rigaud et al., 1995; Rigaud and Lévy, 2003). Detergents with a lower cmc, which form larger micelles, are barely removable by gel filtration or dialysis. Here, the removal can be done using detergent-adsorbent beads. These detergents include Triton-X 100 (Rigaud et al., 1995; Rigaud and Lévy, 2003). Since there are no general protocols available for protein solubilization or reconstitution, they are still accounted for as bottle neck processes (Rigaud et al., 1995; Rigaud and Lévy, 2003; Yanagisawa et al., 2011).

The detergent-mediated reconstitution can also be combined with liposome fabrication methods, such as the double-emulsion approach (Pautot et al., 2003; Yanagisawa et al., 2011). To this end, the solubilized potassium channel protein KcsA was added in the vesicles as well in as the external aqueous phase (Yanagisawa et al., 2011). In both cases, protein reconstitution was detected and the protein was functional (Yanagisawa et al., 2011). During this study, it was observed that not every lipid composition favored the insertion of the target protein. In the case of KcsA, there was no reconstitution detectable when PC lipids were used (Yanagisawa et al., 2011). It was also observed that the reconstitution of KcsA was oriented with the directionality depending on the outside or inside configuration of the protein and the size or the intracellular and extracellular domains (Yanagisawa et al., 2011). KcsA and alpha-hemolysin were successfully reconstituted both from the outer aqueous phase as well as from the inner aqueous solution (Takiguchi et al., 2011; Yanagisawa et al., 2011).

For the reconstitution of KcsA, the detergent DDM was used and due to its high dilution, the detergent concentration was far below the cmc. Nevertheless, detergent molecules can remain in the membranes after successful reconstitution. Since remaining detergent might alter lipid composition and consequently protein behavior, this approach might be unsuitable for certain studies.

## Direct Incorporation of Membrane Proteins into Vesicles in Cell-Free Systems

Another approach to reconstitute transmembrane proteins is to encapsulate the protein translation machinery in vesicles (Figure 2C). The use of cell-free extract to reconstitute transmembrane proteins in liposomes or polymersomes has been successfully reported by various groups (Kalmbach et al., 2007; Murtas et al., 2007; Goren and Fox, 2008; Liguori et al., 2008a,b; Kaneda et al., 2009; Katzen et al., 2009; Kuruma et al., 2009; Maeda et al., 2012; Martino et al., 2012; Liu et al., 2013).

The addition of cDNA coding for the protein of interest allows specific protein synthesis, and it has been shown that membrane proteins can be functionally synthesized and incorporated in the surrounding compartment membrane. Synthesized membrane proteins include pores (Shimizu et al., 2001), channels (Liguori et al., 2008a,b), transporters (Liguori et al., 2008a,b), and receptors (Junge et al., 2011) from eukaryotic and prokaryotic origin (Zanders, 2005). The reconstituted membrane proteins cover sizes from 15 kDa, as the mechanosensitive heptamer protein channel MscL (Madin et al., 2000), up to the 114-kDa transporter MdtB (Zanders, 2005; Liguori et al., 2008a). Also the synthesis of presecretory and integral membrane proteins requiring SecA-dependent translocation, for example, proteins with large periplasmic regions, such as FtsQ, or presecretory proteins, such as OmpA or MtlA (Kim et al., 2006), was reported. An overview of the successful use of cell-free systems is given in Zanders (2005).

There are various cell-free extracts available, but the most commonly used extracts are the whole-wheat germ extract, the *E. coli* extract and the PURESYSTEM. The PURESYSTEM was developed by Kuruma et al. (2008) and includes the chaperone-free *E. coli* translation machinery assembled from purified recombinant components. This defined environment might be beneficial for the functional characterization of proteins, with protein yields of around 6 μg/ml at relative high cost (Kuruma et al., 2008). The wheat germ extract shows stable protein expression for weeks (Zanders, 2005) but is considerably more labor intensive in preparation (Berrier et al., 2004; Sawasaki et al., 2004) than the production of the *E. coli* extract. The *E. coli* assay can be prepared in approximately 1 day (Swartz, 2006; Hovijitra et al., 2009) and shows similar efficiencies as the wheat germ extract with protein yields of around 1–6 mg/ml (Ishihara et al., 2005; Kuruma et al., 2008). While the reaction time of the *E. coli* and the wheat germ extract take between 6 and 24 h for the latter, the reaction time of 2 h is considerably shorter for the PURESYSTEM (Kuruma et al., 2008).

All methods have been used to synthesize and reconstitute troublesome proteins exceeding 100 kDa in size as well as membrane proteins (PURESYSTEM: Kim et al., 2006, wheat germ: Schwarz et al., 2010, and *E. coli*: Madin et al., 2000). Post-translational modifications (Kaiser et al., 2008), such as phosphorylation, prenylation, and glycosylation, as well as the formation of disulfide bonds can be achieved by adding the corresponding enzymes (Kalmbach et al., 2007)_._

The general workflow to achieve protein synthesis in cell-free systems can be summarized as follows: (i) identify the best expression vector compatible with the cell-free expression system (Kuruma et al., 2008). (ii) If immunological detection or fusion with reporter proteins is desired, N-terminal tags showed to generate a higher yield. (iii) After a small-scale optimization step to identify the best expression conditions in expression tests and (iv) a scale-up step, (v) the proteoliposomes can be purified. The estimated time scale from vector design to protein synthesis is around 15 days to 1 month.

An interesting feature of the cell-free systems is the compatibility of the expression systems with some detergents (Madin et al., 2000; Goren and Fox, 2008). A wide range of non-ionic or zwitterionic detergents, Triton X-100, Tween 20, Brij 58p, *n*-dodecyl β-d-maltoside, and CHAPS, were compatible with cell-free synthesis, allowing the expression of proteins in the presence of detergents, while *n*-octyl β-d-glucoside and deoxycholate had an inhibitory effect on protein yield (Madin et al., 2000).

The incorporation of cell-free extract expression systems into vesicles can be achieved using various liposome formation techniques, including natural swelling (Nomura et al., 2003; Kaneda et al., 2009), double-emulsion (Noireaux and Libchaber, 2004; Maeda et al., 2012; Liu et al., 2013), and microfluidic (Martino et al., 2012) approaches.

A drawback of this reconstitution method is the introduction of the complete translation machinery into the lumen of vesicles, which might introduce unwanted complexity to the synthetic system. Also necessary post-translational modifications which might be necessary for the formation of a fully functional protein may not occur, unless the responsible enzymes, if known, are added to the cell-free extract.

## Rehydration of Protein-Containing Agarose

For their reconstitution, solubilized membrane proteins can be dissolved in warm, molten agarose gels (Figure 2B) (Hansen et al., 2013, 2015; Gutierrez and Malmstadt, 2014). In this technique, precipitation of the detergents is prevented at a dilution below the cmc. The gel is spread on a coverslip and partially dehydrated. Since agarose retains a high water content (Horger et al., 2009), it is hypothesized that the proteins do not denature (Hansen et al., 2013). Subsequently, lipid droplets are deposited on top of the gel. Under a stream of nitrogen, the solvent of the droplets is evaporated and the gel can be rehydrated using a protein compatible buffer (Hansen et al., 2013). Upon rehydration of the lipids, protein-containing liposomes form from the surface of the protein-containing agarose. The proteins successfully used in this approach so far were aquaporin-Z, bacteriorhodopsin, and SoPIP2 (Hansen et al., 2013), as well as the glucose transporter GLUT1 (Hansen et al., 2015) and the human serotonin receptor 5-HT1A (Gutierrez and Malmstadt, 2014).

This method is relatively easy but requires a comparably large amount of protein. Moreover, it has been reported that liposomes grown on agarose gels contain agarose in the membrane as well as in the interior (Horger et al., 2009). This introduces a change in the mechanical properties (Lira et al., 2014) of the vesicles, which might introduce artifacts in protein diffusion as well as in enzyme kinetics.

## Using Proteolipobeads for the Reconstitution of Membrane Proteins

Transmembrane protein-coated beads can be applied for the reconstitution of membrane proteins into lipid bilayers (Mirzabekov et al., 2000; Frank et al., 2015). The beads are coated with streptavidin together with a tag or antibody (Figure 2D). The tag allows the purification of the target proteins from detergent-containing cell lysates (Mirzabekov et al., 2000) as well as the coating of the bead with the solubilized transmembrane protein. After addition of detergent-solubilized lipids, the lipids cluster around the protein. The addition of biotinylated lipids, which bind to the streptavidin on the bead, supports the stable and saturated formation of a lipid bilayer around the bead (Mirzabekov et al., 2000). This approach was successfully applied to reconstitute the G-coupled receptor protein CCR5, a seven transmembrane helix protein, into liposomes in the native confirmation and in a uniformly oriented fahion. The detergent is then removed *via* a dialysis step. Here, the non-ionic maltoside detergent cymal was used (Mirzabekov et al., 2000). Throughout the process, the use of paramagnetic beads facilitates buffer changes and the fabrication process (Mirzabekov et al., 2000). A disadvantage of the method is the fixed position of the transmembrane proteins since they are anchored on the bead surface. This reconstitution method is therefore not universally applicable.

## Partial Drying of Liposomes

Another method to achieve protein reconstitution into cell-sized scale vesicles of 1 μm diameter is based on protein-containing bilayers (Girard et al., 2004; Aimon et al., 2011; Fenz et al., 2014). These layers are created by partially drying protein-containing small liposomes. Then, various methods can be employed to form liposomes, including electroformation or swelling (Girard et al., 2004; Aimon et al., 2011; Fenz et al., 2014). A drawback of these techniques is the inevitable rupture and partial drying of the protein-containing vesicles. This is accompanied by the risk of protein denaturation as well as the need for vesicle formation in protein compatible buffers, since mostly high-salt conditions are still required. Electroformation offers a wide range of parameters to fine-tune the vesicle formation process, with a number of protocols available for the use of physiological buffers (Pott et al., 2008). Changes in the electric field (amplitude and frequency), the duration of the protocol, and the swelling buffer can be applied to influence vesicle formation. However, the process of vesicle formation is not yet fully understood (Pott et al., 2008) and side effects, such as lipid-peroxidation (Zhou et al., 2007), or impact of the electric field on the proteins are often not assessable.

Gel-assisted swelling techniques offer a comparably limited range of parameters to improve vesicle production. The use of buffers and lipids involved, as well as the substrate, PVA (Weinberger et al., 2013), agarose (Horger et al., 2009), or others, dominate vesicle size and yield. The processes of vesicle formation in physiological buffers are still not fully understood and require further investigation. The method of proteoliposome formation in the size range greater than one micron is therefore still limited by the available protocols.

## Peptide-Induced Fusion

Membrane protein incorporation into larger vesicles can also be achieved by the fusion of vesicles. Besides detergent-mediated fusion (as already discussed above), other, non-detergent-mediated fusion techniques have been elaborated, including the work of Kahya et al. (2001). Here, the fusogenic peptide WAE has been used to initiate vesicle fusion. Liposomes were formed out of DOPC:chol/PE-PDP (3.5:1.5:0.25), and the WAE peptide was subsequently covalently attached to the vesicles (Pécheur et al., 1997, 1999; Kahya et al., 2001). Larger vesicles, with positively charged lipids, a mixture of DOPC:DOPE:SAINT-2 (10:3:1.3), were used as peptide target. Under these conditions, fusion events were observed and the method was successfully used to reconstitute bacteriorhodopsin (Kahya et al., 2001) as well as a complex of the seven-helix photoreceptor NpSRII and its cognate transducer NpHtrII, with the latter containing two transmembrane α-helices and a large cytoplasmic domain (Kriegsmann et al., 2009).

## Mechanical and Spontaneous Insertion of Membrane Proteins

In other reconstitution techniques, solubilized proteins are added to the already formed liposomes. Defects in the liposome membrane are either induced by sonification pulses (Rigaud and Lévy, 2003), electrical pulses (Rigaud and Lévy, 2003), or freeze-thawing steps (Kammerer et al., 1998; Rigaud and Lévy, 2003). The drawback of these techniques is that mechanical stress is imposed on the solubilized proteins, which can lead to denaturation or low reconstitution rates (Rigaud and Lévy, 2003).

Some classes of proteins, e.g., cytochromes, bacteriorhodopsin and F_0_F_1_-ATPases, and porins (Elani et al., 2013, 2014), show spontaneous incorporation into lipid bilayers without the addition of any detergent. Nevertheless, a certain lipid composition of mostly acidic lipids (Eytan and Broza, 1978), as well as vesicles of small size (Eytan and Broza, 1978; Eytan, 1982), is required for spontaneous insertion of these transmembrane proteins. This process has been examined more closely. Jain and Zakim (1987) have revealed that defects in the membrane associated with amphiphilic contaminants as cholesterols, short-chain lipids, and others are facilitating spontaneous insertion.

## Conclusion

Artificial vesicles are a versatile resource to establish compartmentation in synthetic biochemical nanoreactors. To this end, compartmentation can either be achieved by immobilizing artificial liposome or polymersome vesicles to the matrix of microfluidic reactors or by generating nested or concatenated vesicular systems such as vesosomes.

In order to maximize yield of biochemical processes inside vesicular nanoreactors, it is indispensable to control the exchange of substrates and products across the membranes between the reactor compartments. Technically, it is possible to reconstitute entire metabolic pathways into liposomes or polymersomes, and by the use of vesosomes or immobilized vesicles, it seems even feasible to mimic the natural compartmentation of these pathways in synthetic systems. However, the exchange of substances across membrane boundaries needs to be highly specific to make compartmented reconstituted biochemical pathways work.

Exchange of substances across membranes can either be accomplished by utilizing lipid and coblock polymers that change permeability in response to physicochemical triggers such as heat, redox potential, or pH. However, only unspecific mixing of contents can be achieved by altering the permeability of the membrane boundary. The incorporation of unspecific protein pores into the membranes of vesicle compartmented reactors, e.g., porins or α-hemolysin, allows for a more selective exchange of low molecular weight substances but still permits the passage of a variety of intermediates that are similar in charge and/or structure. Ultimately, the reconstitution of specific membrane transporters that only allow the passage of individual substrates is necessary for the functional reconstitution of compartmented biochemical pathways. As outlined in this review, diverse approaches can be undertaken to achieve successful reconstitution of membrane transport proteins into artificial membranes. Since the optimal conditions for successful reconstitution are quite specific for each individual transport protein and can hardly be transferred to other candidates, we have taken care to summarize the available techniques for membrane transporter reconstitution. We believe that the integration of metabolite transporters into vesicle-based nanoreactors will largely advance bottom-up approaches in the development of compartmented protocells in the future.

## Author Contributions

CS, AL, and LV wrote the article. NB, VS, and LV conceptualized the article.

## Conflict of Interest Statement

The authors declare that the research was conducted in the absence of any commercial or financial relationships that could be construed as a potential conflict of interest.

## Abbreviations

DOPC, 1,2-dioleoyl-*sn*-glycero-3-phosphocholine; DPPC, 1,2-dipalmitoyl-*sn*-glycero-3-phosphocholine; PB-*b*-PEO, polybutadiene-*b*-poly(ethylene oxide); PEG-*b*-PSBA, poly(ethylene glycol)-*b*-poly(styrene boronic acid); PEO–PPO–PEO, poly(ethylene oxide)–poly(propylene oxide)–poly(ethylene oxide); PEtOz-PDMS-PEtOz, poly(2-ethyl-2-oxazoline)-*b*-poly(dimethylsiloxane)-*b*-poly(2-ethyl-2-oxazoline); PMOXA–PDMS–PMOXA, poly(2-methyloxazoline)–poly(dimethylsiloxane)–poly(2-methyl-oxazoline); PS-*b*-PDMAEMA, polystyrene-*b*-poly(*N*,*N*-dimethylaminoethyl methacrylate); PS-*b*–PIAT, polystyrene–poly(l-isocyanoalanine(2-thiophen-3-yl-ethyl)amide), aka polystyrene-*b*-poly(3-(isocyano-lalanyl-amino-ethyl)-thiophene); PVFc-*b*-P2VP, polyvinylferrocene-*b*-poly(2-vinylpyridine).

## References

[B1] Aimon S. Manzi J. Schmidt D. Poveda Larrosa J. A. Bassereau P. Toombes G. E. (2011). Functional reconstitution of a voltage-gated potassium channel in giant unilamellar vesicles. PLoS ONE 6:e25529. 10.1371/journal.pone.0025529 21998666 PMC3188570

[B2] Andrès C. Agne B. Kessler F. (2010). The TOC complex: preprotein gateway to the chloroplast. Biochim. Biophys. Acta 1803, 715–723. 10.1016/j.bbamcr.2010.03.004 20226817

[B3] Bauer M. F. Hofmann S. Neupert W. Brunner M. (2000). Protein translocation into mitochondria: the role of TIM complexes. Trends Cell Biol. 10, 25–31. 10.1016/S0962-8924(99)01684-0 10603473

[B4] Bermudez H. Brannan A. K. Hammer D. A. Bates F. S. Discher D. E. (2002). Molecular weight dependence of polymersome membrane structure, elasticity, and stability. Macromolecules 35, 8203–8208. 10.1021/ma020669l

[B5] Berrier C. Park K. H. Abes S. Bibonne A. Betton J. M. Ghazi A. (2004). Cell-free synthesis of a functional ion channel in the absence of a membrane and in the presence of detergent. Biochemistry 43, 12585–12591. 10.1021/bi049049y 15449948

[B6] Binder W. H. (2008). Polymer-induced transient pores in lipid membranes. Angew. Chem. Int. Ed. Engl. 47, 3092–3095. 10.1002/anie.200800269 18338350

[B7] Bolinger P.-Y. Stamou D. Vogel H. (2004). Integrated nanoreactor systems: triggering the release and mixing of compounds inside single vesicles. J. Am. Chem. Soc. 126, 8594–8595. 10.1021/ja049023u 15250679

[B8] Bolinger P.-Y. Stamou D. Vogel H. (2008). An integrated self-assembled nanofluidic system for controlled biological chemistries. Angew. Chem. Int. Ed. Engl. 47, 5544–5549. 10.1002/anie.200801606 18613154

[B9] Choi H.-J. Montemagno C. D. (2005). Artificial organelle: ATP synthesis from cellular mimetic polymersomes. Nano Lett. 5, 2538–2542. 10.1021/nl051896e 16351211

[B10] Cowan S. W. Garavito R. M. Jansonius J. N. Jenkins J. A. Karlsson R. König N. (1995). The structure of OmpF porin in a tetragonal crystal form. Structure 3, 1041–1050. 10.1016/S0969-2126(01)00240-4 8589999

[B11] De Hoog H. P. M. Arends I. W. C. E. Rowan A. E. Cornelissen J. J. L. M. Nolte R. J. M. (2010). A hydrogel-based enzyme-loaded polymersome reactor. Nanoscale 2, 709–716. 10.1039/b9nr00325h 20648315

[B12] Dezi M. Di Cicco A. Bassereau P. Levy D. (2013). Detergent-mediated incorporation of transmembrane proteins in giant unilamellar vesicles with controlled physiological contents. Proc. Natl. Acad. Sci. U.S.A. 110, 7276–7281. 10.1073/pnas.1303857110 23589883 PMC3645586

[B13] Discher D. E. Eisenberg A. (2002). Polymer vesicles. Science 297, 967–973. 10.1126/science.1074972 12169723

[B14] Economou A. (1999). Following the leader: bacterial protein export through the Sec pathway. Trends Microbiol. 7, 315–320. 10.1016/S0966-842X(99)01555-3 10431204

[B15] Elani Y. Gee A. Law R. V. Ces O. (2013). Engineering multi-compartment vesicle networks. Chem. Sci. 4, 3332–3338. 10.1039/c3sc51164b

[B16] Elani Y. Law R. V. Ces O. (2014). Vesicle-based artificial cells as chemical microreactors with spatially segregated reaction pathways. Nat. Commun. 5, 5305. 10.1038/ncomms6305 25351716

[B17] Engelman D. M. Steitz T. A. (1981). The spontaneous insertion of proteins into and across membranes: the helical hairpin hypothesis. Cell 23, 411–422. 10.1016/0092-8674(81)90136-7 7471207

[B18] Eytan G. D. (1982). Use of liposomes for reconstitution of biological functions. Biochim. Biophys. Acta 694, 185–202. 10.1016/0304-4157(82)90024-7 6753932

[B19] Eytan G. D. Broza R. (1978). Role of charge and fluidity in the incorporation of cytochrome oxidase into liposomes. J. Biol. Chem. 253, 3196–3202. 205542

[B20] Fenz S. F. Sachse R. Schmidt T. Kubick S. (2014). Cell-free synthesis of membrane proteins: tailored cell models out of microsomes. Biochim. Biophys. Acta 1838, 1382–1388. 10.1016/j.bbamem.2013.12.009 24370776

[B21] Frank P. Siebenhofer B. Hanzer T. Geiss A. F. Schadauer F. Reiner-Rozman C. (2015). Proteo-lipobeads for the oriented encapsulation of membrane proteins. Soft Matter 11, 2906–2908. 10.1039/c4sm02646b 25763882 PMC4387127

[B22] Girard P. Pécréaux J. Lenoir G. Falson P. Rigaud J. L. Bassereau P. (2004). A new method for the reconstitution of membrane proteins into giant unilamellar vesicles. Biophys. J. 87, 419–429. 10.1529/biophysj.104.040360 15240476 PMC1304363

[B23] Goren M. A. Fox B. G. (2008). Wheat germ cell-free translation, purification, and assembly of a functional human stearoyl-CoA desaturase complex. Protein Expr. Purif. 62, 171–178. 10.1016/j.pep.2008.08.002 18765284 PMC2586813

[B24] Graff A. Winterhalter M. Meier W. (2001). Nanoreactors from polymer-stabilized liposomes. Langmuir 17, 919–923. 10.1021/la001306m

[B25] Gutensohn M. Fan E. Frielingsdorf S. Hanner P. Hou B. Hust B. (2006). Toc, Tic, Tat et al.: structure and function of protein transport machineries in chloroplasts. J. Plant Physiol. 163, 333–347. 10.1016/j.jplph.2005.11.009 16386331

[B26] Gutierrez M. G. Malmstadt N. (2014). Human serotonin receptor 5-HT 1A preferentially segregates to the liquid disordered phase in synthetic lipid bilayers. J. Am. Chem. Soc. 136, 13530–13533. 10.1021/ja507221m 25211019 PMC4183657

[B27] Hansen J. S. Elbing K. Thompson J. R. Malmstadt N. Lindkvist-Petersson K. (2015). Glucose transport machinery reconstituted in cell models. Chem. Commun. 51, 2316–2319. 10.1039/c4cc08838g 25562394 PMC4308475

[B28] Hansen J. S. Thompson J. R. Hélix-Nielsen C. Malmstadt N. (2013). Lipid directed intrinsic membrane protein segregation. J. Am. Chem. Soc. 135, 17294–17297. 10.1021/ja409708e 24180248 PMC3886709

[B29] Horger K. S. Estes D. J. Capone R. Mayer M. (2009). Films of agarose enable rapid formation of giant liposomes in solutions of physiologic ionic strength. J. Am. Chem. Soc. 131, 1810–1819. 10.1021/ja805625u 19154115 PMC2757642

[B30] Hovijitra N. T. Wuu J. J. Peaker B. Swartz J. R. (2009). Cell-free synthesis of functional aquaporin Z in synthetic liposomes. Biotechnol. Bioeng. 104, 40–49. 10.1002/bit.22385 19557835

[B31] Ishihara G. Goto M. Saeki M. Ito K. Hori T. Kigawa T. (2005). Expression of G protein coupled receptors in a cell-free translational system using detergents and thioredoxin-fusion vectors. Protein Expr. Purif. 41, 27–37. 10.1016/j.pep.2005.01.013 15802218

[B32] Jain M. K. Zakim D. (1987). The spontaneous incorporation of proteins into preformed bilayers. Biochim. Biophys. Acta 906, 33–68. 10.1016/0304-4157(87)90004-9 3032257

[B33] Junge F. Haberstock S. Roos C. Stefer S. Proverbio D. Dötsch V. (2011). Advances in cell-free protein synthesis for the functional and structural analysis of membrane proteins. New Biotechnol. 28, 262–271. 10.1016/j.nbt.2010.07.002 20637904

[B34] Kagawa Y. Racker E. (1971). Partial resolution of the enzymes catalyzing oxidative phosphorylation XXV. Reconstitution of vesicles catalyzing ^32^P_i_-adenosine triphosphate exchange. J. Biol. Chem. 246, 5477–5487.

[B35] Kahya N. Pécheur E. I. de Boeij W. P. Wiersma D. A. Hoekstra D. (2001). Reconstitution of membrane proteins into giant unilamellar vesicles via peptide-induced fusion. Biophys. J. 81, 1464–1474. 10.1016/S0006-3495(01)75801-8 11509360 PMC1301625

[B36] Kaiser L. Graveland-Bikker J. Steuerwald D. Vanberghem M. Herlihy K. Zhang S. (2008). Efficient cell-free production of olfactory receptors: detergent optimization, structure, and ligand binding analyses. Proc. Natl. Acad. Sci. U.S.A. 105, 15726–15731. 10.1073/pnas.0804766105 18840687 PMC2572932

[B37] Kalmbach R. Kalmbach R. Chizhov I. Schumacher M. C. Friedrich T. Bamberg E. (2007). Functional cell-free synthesis of a seven helix membrane protein: *in situ* insertion of bacteriorhodopsin into liposomes. J. Mol. Biol. 371, 639–648. 10.1016/j.jmb.2007.05.087 17586523

[B38] Kammerer B. Fischer K. Hilpert B. Schubert S. Gutensohn M. Weber A. (1998). Molecular characterization of a carbon transporter in plastids from heterotrophic tissues: the glucose 6-phosphate/phosphate antiporter. Plant Cell 10, 105–117. 10.2307/3870632 9477574 PMC143937

[B39] Kaneda M. Nomura S. M. Ichinose S. Kondo S. Nakahama K. Akiyoshi K. (2009). Direct formation of proteo-liposomes by in vitro synthesis and cellular cytosolic delivery with connexin-expressing liposomes. Biomaterials 30, 3971–3977. 10.1016/j.biomaterials.2009.04.006 19423159

[B40] Katzen F. Peterson T. C. Kudlicki W. (2009). Membrane protein expression: no cells required. Trends Biotechnol. 27, 455–460. 10.1016/j.tibtech.2009.05.005 19616329

[B41] Khan I. Khan M. Umar M. N. Oh D. H. (2015). Nanobiotechnology and its applications in drug delivery system: a review. IET Nanobiotechnol. 9, 396–400. 10.1049/iet-nbt.2014.0062 26647817

[B42] Kim K. T. Cornelissen J. J. L. M. Nolte R. J. M. van Hest J. C. M. (2009). A polymersome nanoreactor with controllable permeability induced by stimuli-responsive block copolymers. Adv. Mater. 21, 2787–2791. 10.1002/adma.200900300

[B43] Kim T.-W. Keum J. W. Oh I. S. Choi C. Y. Park C. G. Kim D. M. (2006). Simple procedures for the construction of a robust and cost-effective cell-free protein synthesis system. J. Biotechnol. 126, 554–561. 10.1016/j.jbiotec.2006.05.014 16797767

[B44] Kriegsmann J. Gregor I. von der Hocht I. Klare J. Engelhard M. Enderlein J. (2009). Translational diffusion and interaction of a photoreceptor and its cognate transducer observed in giant unilamellar vesicles by using dual-focus FCS. Chembiochem 10, 1823–1829. 10.1002/cbic.200900251 19551796

[B45] Kuiper S. M. Nallani M. Vriezema D. M. Cornelissen J. J. van Hest J. C. M. Nolte R. J. (2008). Enzymes containing porous polymersomes as nano reaction vessels for cascade reactions. Org. Biomol. Chem. 6, 4315–4318. 10.1039/b811196k 19005589

[B46] Kuruma Y. Nishiyama K. Shimizu Y. Müller M. Ueda T. (2008). Development of a minimal cell-free translation system for the synthesis of presecretory and integral membrane proteins. Biotechnol. Prog. 21, 1243–1251. 10.1021/bp049553u 16080708

[B47] Kuruma Y. Stano P. Ueda T. Luisi P. L. (2009). A synthetic biology approach to the construction of membrane proteins in semi-synthetic minimal cells. Biochim. Biophys. Acta 1788, 567–574. 10.1016/j.bbamem.2008.10.017 19027713

[B48] Lee J. S. Feijen J. (2012). Polymersomes for drug delivery: design, formation and characterization. J. Control. Release 161, 473–483. 10.1016/j.jconrel.2011.10.005 22020381

[B49] Liguori L. Marques B. Lenormand J.-L. (2008a). A bacterial cell-free expression system to produce membrane proteins and proteoliposomes: from cDNA to functional assay. Curr. Protoc. Protein Sci. 5, 22. 10.1002/0471140864.ps0522s54 19016436

[B50] Liguori L. Marques B. Villegas-Mendez A. Rothe R. Lenormand J.-L. (2008b). Liposomes-mediated delivery of pro-apoptotic therapeutic membrane proteins. J. Control. Release 126, 217–227. 10.1016/j.jconrel.2007.12.004 18234390

[B51] Lira R. B. Dimova R. Riske K. A. (2014). Giant unilamellar vesicles formed by hybrid films of agarose and lipids display altered mechanical properties. Biophys. J. 107, 1609–1619. 10.1016/j.bpj.2014.08.009 25296313 PMC4190656

[B52] Liu Y.-J. Hansen G. P. R. Venancio-Marques A. Baigl D. (2013). Cell-free preparation of functional and triggerable giant proteoliposomes. Chembiochem 14, 2243–2247. 10.1002/cbic.201300501 24115581

[B53] Madin K. Sawasaki T. Ogasawara T. Endo Y. (2000). A highly efficient and robust cell-free protein synthesis system prepared from wheat embryos: plants apparently contain a suicide system directed at ribosomes. Proc. Natl. Acad. Sci. U.S.A. 97, 559–564. 10.1073/pnas.97.2.559 10639118 PMC15369

[B54] Maeda Y. T. Nakadai T. Shin J. Uryu K. Noireaux V. Libchaber A. (2012). Assembly of MreB filaments on liposome membranes: a synthetic biology approach. ACS Synth. Biol. 1, 53–59. 10.1021/sb200003v 23651045

[B55] Marguet M. Edembe L. Lecommandoux S. (2012). Polymersomes in polymersomes: multiple loading and permeability control. Angew. Chem. Int. Ed. Engl. 124, 1199–1202. 10.1002/ange.201106410 22190263

[B56] Marsh D. (2012). Thermodynamics of phospholipid self-assembly. Biophys. J. 102, 1079–1087. 10.1016/j.bpj.2012.01.049 22404930 PMC3296042

[B57] Martino C. Kim S. H. Horsfall L. Abbaspourrad A. Rosser S. J. Cooper J. (2012). Protein expression, aggregation, and triggered release from polymersomes as artificial cell-like structures. Angew. Chem. Int. Ed. Engl. 51, 6416–6420. 10.1002/anie.201201443 22644870

[B58] Meier W. Nardin C. Winterhalter M. (2000). Reconstitution of channel proteins in (polymerized) ABA triblock copolymer membranes. Angew. Chem. Int. Ed. Engl. 39, 4599–4602. 10.1002/1521-3773(20001215)39:24<4599::AID-ANIE4599>3.0.CO;2-Y 11169683

[B59] Mirzabekov T. Kontos H. Farzan M. Marasco W. Sodroski J. (2000). Paramagnetic proteoliposomes containing a pure, native, and oriented seventransmembrane segment protein, CCR5. Nat. Biotechnol. 18, 649–654. 10.1038/76501 10835604

[B60] Montes L.-R. Alonso A. Goñi F. M. Bagatolli L. A. (2007). Giant unilamellar vesicles electroformed from native membranes and organic lipid mixtures under physiological conditions. Biophys. J. 93, 3548–3554. 10.1529/biophysj.107.116228 17704162 PMC2072068

[B61] Murtas G. Kuruma Y. Bianchini P. Diaspro A. Luisi P. L. (2007). Protein synthesis in liposomes with a minimal set of enzymes. Biochem. Biophys. Res. Commun. 363, 12–17. 10.1016/j.bbrc.2007.07.201 17850764

[B62] Nallani M. Andreasson-Ochsner M. Tan C. W. Sinner E. K. Wisantoso Y. Geifman-Shochat S. (2011). Proteopolymersomes: *in vitro* production of a membrane protein in polymersome membranes. Biointerphases 6, 153–157. 10.1116/1.3644384 22239807

[B63] Nardin C. Thoeni S. Widmer J. Winterhalter M. Meier W. (2000). Nanoreactors based on (polymerized) ABA-triblock copolymer vesicles. Chem. Commun. 2000, 1433–1434. 10.1039/b004280n

[B64] Nardin C. Widmer J. Winterhalter M. Meier W. (2001). Amphiphilic block copolymer nanocontainers as bioreactors. Eur. Phys. J. E 4, 403–410. 10.1007/s101890170095

[B65] Noireaux V. Libchaber A. (2004). A vesicle bioreactor as a step toward an artificial cell assembly. Proc. Natl. Acad. Sci. U.S.A. 101, 17669–17674. 10.1073/pnas.0408236101 15591347 PMC539773

[B66] Nomura S. M. Nomura S. M. Tsumoto K. Hamada T. Akiyoshi K. Nakatani Y. (2003). Gene expression within cell-sized lipid vesicles. Chembiochem 4, 1172–1175. 10.1002/cbic.200300630 14613108

[B67] Ohya Y. Takahashi A. Nagahama K. (2011). Biodegradable polymeric assemblies for biomedical materials. Adv. Polym. Sci. 247, 65–114. 10.1007/12_2011_160

[B68] Ollivon M. Lesieur S. Grabielle-Madelmont C. Paternostre M. (2000). Vesicle reconstitution from lipid-detergent mixed micelles. Biochim. Biophys. Acta 1508, 34–50. 10.1016/S0304-4157(00)00006-X 11090817

[B69] Paleos C. M. Tsiourvas D. Sideratou Z. (2012). Preparation of multicompartment lipid-based systems based on vesicle interactions. Langmuir 28, 2337–2346. 10.1021/la2027187 21988476

[B70] Pautot S. Frisken B. J. Weitz D. A. (2003). Production of unilamellar vesicles using an inverted emulsion. Langmuir 19, 2870–2879. 10.1021/la026100v

[B71] Pécheur E. I. Hoekstra D. Sainte-Marie J. Maurin L. Bienvenüe A. Philippot J. R. (1997). Membrane anchorage brings about fusogenic properties in a short synthetic peptide. Biochemistry 36, 3773–3781. 10.1021/bi9622128 9092806

[B72] Pécheur E. I. Sainte-Marie J. Bienvenüe A. Hoekstra D. (1999). Peptides and membrane fusion: towards an understanding of the molecular mechanism of protein-induced fusion. J. Membr. Biol. 167, 1–17. 10.1007/s002329900466 9878070

[B73] Peters R. J. Marguet M. Marais S. Fraaije M. W. van Hest J. C. M. Lecommandoux S. (2014). Cascade reactions in multicompartmentalized polymersomes. Angew. Chem. Int. Ed. Engl. 126, 150–154. 10.1002/anie.201308141 24254810

[B74] Pott T. Bouvrais H. Méléard P. (2008). Giant unilamellar vesicle formation under physiologically relevant conditions. Chem. Phys. Lipids 154, 115–119. 10.1016/j.chemphyslip.2008.03.008 18405664

[B75] Rapoport T. A. Goder V. Heinrich S. U. Matlack K. E. S. (2004). Membrane-protein integration and the role of the translocation channel. Trends Cell Biol. 14, 568–575. 10.1016/j.tcb.2004.09.002 15450979

[B76] Rigaud J.-L. Lévy D. (2003). Reconstitution of membrane proteins into liposomes. Meth. Enzymol. 372, 65–86. 10.1016/S0076-6879(03)72004-7 14610807

[B77] Rigaud J.-L. Paternostre M. T. Bluzat A. (1988). Mechanisms of membrane protein insertion into liposomes during reconstitution procedures involving the use of detergents. 2. Incorporation of the light-driven proton pump bacteriorhodopsin. Biochemistry 27, 2677–2688. 10.1021/bi00408a007 3401443

[B78] Rigaud J.-L. Pitard B. Levy D. (1995). Reconstitution of membrane proteins into liposomes: application to energy-transducing membrane proteins. Biochim. Biophys. Acta 1231, 223–246. 10.1016/0005-2728(95)00091-V 7578213

[B79] Rodríguez-García R. Mell M. López-Montero I. Netzel J. Hellweg T. Monroy F. (2011). Polymersomes: smart vesicles of tunable rigidity and permeability. Soft Matter 7, 1532–1542. 10.1039/c0sm00823k

[B80] Ruta V. Jiang Y. Lee A. Chen J. MacKinnon R. (2003). Functional analysis of an archaebacterial voltage-dependent K+ channel. Nature 422, 180–185. 10.1038/nature01473 12629550

[B81] Sawasaki T. Hasegawa Y. Morishita R. Seki M. Shinozaki K. Endo Y. (2004). Genome-scale, biochemical annotation method based on the wheat germ cell-free protein synthesis system. Phytochemistry 65, 1549–1555. 10.1016/j.phytochem.2004.04.023 15276451

[B82] Schwarz D. Daley D. Beckhaus T. Dötsch V. Bernhard F. (2010). Cell-free expression profiling of *E. coli* inner membrane proteins. Proteomics 10, 1762–1779. 10.1002/pmic.200900485 20198639

[B83] Seddon A. M. Curnow P. Booth P. J. (2004). Membrane proteins, lipids and detergents: not just a soap opera. Biochim. Biophys. Acta 1666, 105–117. 10.1016/j.bbamem.2004.04.011 15519311

[B84] Shimizu Y. Inoue A. Tomari Y. Suzuki T. Yokogawa T. Nishikawa K. (2001). Cell-free translation reconstituted with purified components. Nat. Biotechnol. 19, 751–755. 10.1038/90802 11479568

[B85] Siti W. de Hoog H.-P. Fischer O. Shan W. S. Tomczak N. Nallani M. (2014). An intercompartmental enzymatic cascade reaction in channel-equipped polymersome-in-polymersome architectures. J. Mater. Chem. B 2, 2733–2737. 10.1039/c3tb21849j 32261439

[B86] Smart T. Lomas H. Massignani M. Flores-Merino M. V. Perez L. R. Battaglia G. (2008). Block copolymer nanostructures. Nano Today 3, 38–46. 10.1016/S1748-0132(08)70043-4

[B87] Staff R. H. Gallei M. Landfester K. Crespy D. (2014). Hydrophobic nanocontainers for stimulus-selective release in aqueous environments. Macromolecules 47, 4876–4883. 10.1021/ma501233y

[B88] Steinberg-Yfrach G. Rigaud J. L. Durantini E. N. Moore A. L. Gust D. Moore T. A. (1998). Light-driven production of ATP catalysed by F0F1-ATP synthase in an artificial photosynthetic membrane. Nature 392, 479. 10.1038/33116 9548252

[B89] Swartz J. (2006). Developing cell-free biology for industrial applications. J. Ind. Microbiol. Biotechnol. 33, 476–485. 10.1007/s10295-006-0127-y 16761165

[B90] Takiguchi K. Negishi M. Tanaka-Takiguchi Y. Homma M. Yoshikawa K. (2011). Transformation of actoHMM assembly confined in cell-sized liposome. Langmuir 27, 11528–11535. 10.1021/la2016287 21819144 PMC3171996

[B91] Thambi T. Park J. H. Lee D. S. (2016). Stimuli-responsive polymersomes for cancer therapy. Biomater. Sci. 4, 55–69. 10.1039/C5BM00268K 26456625

[B92] Torchilin V. Weissig V. (2003). Liposomes: A Practical Approach. London, UK: Oxford University Press.

[B93] Ullrich M. Hanuš J. Štěphánek F. (2015). Remote control of enzymatic reaction in compartmentalized microparticles: a system for the delivery of unstable actives. Chem. Eng. Sci. 125, 191–199. 10.1016/j.ces.2014.06.020

[B94] Van Hoogevest P. de Gier J. de Kruijff B. (1984). Determination of the size of the packing defects in dimyristoylphosphatidylcholine bilayers, present at the phase transition temperature. FEBS Lett. 171, 160–164. 10.1016/0014-5793(84)80479-2

[B95] Vriezema D. M. Garcia P. M. L. Sancho Oltra N. Hatzakis N. S. Kuiper S. M. Nolte R. J. M. (2007). Positional assembly of enzymes in polymersome nanoreactors for cascade reactions. Angew. Chem. Int. Ed. Engl. 119, 7522–7526. 10.1002/ange.200701125 17705203

[B96] Walker S. A. Kennedy M. T. Zasadzinski J. A. (1997). Encapsulation of bilayer vesicles by self-assembly. Nature 387, 61–64. 10.1038/387061a0 9139822

[B97] Weinberger A. Tsai F. C. Koenderink G. H. Schmidt T. F. Itri R. Meier W. (2013). Gel-assisted formation of giant unilamellar vesicles. Biophys. J. 105, 154–164. 10.1016/j.bpj.2013.05.024 23823234 PMC3699747

[B98] Wickner W. T. Lodish H. F. (1985). Multiple mechanisms of protein insertion into and across membranes. Science 230, 400–407. 10.1126/science.4048938 4048938

[B99] Yanagisawa M. Iwamoto M. Kato A. Yoshikawa K. Oiki S. (2011). Oriented reconstitution of a membrane protein in a giant unilamellar vesicle: experimental verification with the potassium channel KcsA. J. Am. Chem. Soc. 133, 11774–11779. 10.1021/ja2040859 21702488

[B100] Zanders E. D. (2005). Chemical Genomics: Reviews and Protocols. New York, USA: Humana Press.

[B101] Zhao L. Li N. Wang K. Shi C. Zhang L. Luan Y. (2014). A review of polypeptide-based polymersomes. Biomaterials 35, 1284–1301. 10.1016/j.biomaterials.2013.10.063 24211077

[B102] Zhou Y. Berry C. K. Storer P. A. Raphael R. M. (2007). Peroxidation of polyunsaturated phosphatidyl-choline lipids during electroformation. Biomaterials 28, 1298–1306. 10.1016/j.biomaterials.2006.10.016 17107709

